# Embryonic Thermal Programming and Dietary Baicalein Supplementation Post-Hatch: Effects on Broiler Adipose Tissue Deposition

**DOI:** 10.3390/ani14243563

**Published:** 2024-12-10

**Authors:** Usman Sulaiman, Reagan Vaughan, Paul Siegel, Dongmin Liu, Elizabeth Gilbert, Mark Cline

**Affiliations:** 1 School of Animal Sciences, Virginia Polytechnic Institute and State University, Blacksburg, VA 24061, USA; usulaiman@vt.edu (U.S.); pbsiegel@vt.edu (P.S.); 2 Department of Human Nutrition, Foods and Exercise, Virginia Polytechnic Institute and State University, Blacksburg, VA 24061, USA; reagansvaughan@vt.edu (R.V.); doliu@vt.edu (D.L.); 3 School of Neuroscience, Virginia Polytechnic Institute and State University, Blacksburg, VA 24061, USA; egilbert@vt.edu

**Keywords:** adipose tissue, baicalein, embryonic heat conditioning, heat stress, broilers, mRNA expression, global warming

## Abstract

Optimization of growth performance and fat metabolism in broilers are critical for meat quality and overall production efficiency. This experiment investigated the effects of dietary baicalein supplementation and embryonic heat conditioning (EHC) on the growth performance and adipose tissue metabolism of 10-day old broilers. Fertile eggs were divided into control and EHC groups, with EHC eggs exposed to intermittent heating (39.5 °C) from day 7 to day 16 of incubation. Hatched chicks were further divided into four groups: CC (control control), CT (control treatment with baicalein), EC (embryonic heat control), and ET (embryonic heat treatment with baicalein), and were fed ad libitum. On day 10 post-hatch, blood and adipose tissue samples were collected for analysis. *C/EBPα* mRNA was lower in the ET group compared to the EC group and higher in the CT group compared to the CC group. *PPARγ* and *HSL* mRNAs were elevated in both the ET and CT groups relative to their controls. Additionally, plasma non-esterified fatty acid (NEFA) levels were significantly higher in the CT group compared to the CC group. These results indicate that baicalein supplementation, particularly when combined with embryonic heat conditioning, can modulate fat metabolism and potentially improve the growth performance of broilers, thereby offering insights into strategies for enhancing poultry production.

## Simple Summary

Poultry production is crucial for meeting global food demands, and optimizing the growth and health of broiler chickens is a key focus for researchers. This experiment explored how a natural compound, baicalein, combined with specific heat exposure during egg incubation, affects the growth and fat metabolism of broiler chickens. The experiment showed that chickens receiving both heat treatment during embryonic development and baicalein treatment after hatching had lower levels of a fat-formation gene and higher activity of genes related to fat breakdown, leading to healthier fat tissue. These findings suggest that such combined treatments could be a promising strategy to enhance poultry growth and health, which could benefit farmers by improving meat quality and production efficiency.

## 1. Introduction

Adipose tissue is a vital organ in poultry and plays a crucial role in energy metabolism, body weight regulation, and overall physiological homeostasis [1]. This tissue not only serves as the primary site for lipid storage but also functions as an endocrine organ, secreting various hormones and cytokines known as adipokines that influence metabolic processes throughout the body [2,3]. Therefore, the regulation of adipose tissue development and function is central to maintaining energy balance and preventing metabolic disorders, particularly in the context of modern poultry production systems where rapid growth and high feed efficiency are prioritized [4,5].

In the poultry industry, optimizing growth rates and meat yield is a key objective, driven by the increasing demand for poultry products worldwide [6]. Genetic selection has enabled the production of broiler chickens with remarkable growth potential, allowing them to reach market weight in a shorter period [7]. However, this genetic advancement has come with unintended consequences, notably an increased propensity for excessive fat deposition [8]. While a certain level of adipose tissue is necessary for energy storage and insulation, excessive fat accumulation can lead to negative outcomes, including reduced feed efficiency, compromised meat quality, and increased risk of metabolic disorders [9]. These issues pose challenges not only for the health and welfare of birds but also for the economic sustainability of poultry production [10].

Thermoregulation is another critical aspect of avian physiology that directly impacts growth performance and metabolic health. The poultry industry has long been interested in manipulating the thermal environment of avian embryos to enhance their post-hatch performance [11]. One such approach is embryonic heat conditioning (EHC). This technique involves exposing embryos to elevated temperatures during specific windows of development to improve the thermoregulatory capacity of hatchlings [12]. Studies have shown that EHC leads to the development of a better phenotype regarding thermoregulation later in life, as it helps establish a higher heat threshold. EHC has also been reported to help with stress management, increased immunological state, and cross-tolerance [13,14]. Furthermore, EHC has been found to lead to the upregulation of lipolytic indicators in broiler adipose tissue post-hatch [15].

Baicalein is a flavonoid compound found in a variety of plants, including the root of Chinese skullcap (*Scutellaria baicalensis*) [16]. It has been shown to have anti-inflammatory, antioxidant, antibacterial, antiviral, and anticancer effects in animal and cell culture studies [17,18,19,20,21]. The effect of dietary supplementation with baicalein on adipose tissue deposition in broiler chickens is an area of active research. However, studies on this topic are limited, and further research is needed. Xiao et al. (2021) reported that dietary supplementation with baicalein led to a reduction in the abdominal fat mass of broiler chicks on day 6 post-hatch [22]. Consistent with this finding, it was reported that adipogenic gene expression was lower in the treatment groups compared to the control.

Amaz et al. (2024) investigated how embryonic thermal manipulation and post-hatch baicalein supplementation influenced broilers, focusing on liver metabolism and muscle growth under heat stress. This study found that these interventions improved gene expression related to metabolic and muscle functions, highlighting the potential benefits of embryonic thermal manipulation and baicalein for broiler health [23].

This experiment aimed to investigate the effects of EHC and dietary baicalein supplementation post-hatch on broiler adipose tissue deposition by measuring the changes in gene expression of adipogenic factors using qPCR. Additionally, this study evaluated non-esterified fatty acid (NEFA) concentrations in chicks in all treatments. In contrast to a previous paper involving dietary baicalein, in which broilers were sampled on day 6 post-hatch, in this experiment, samples were collected on day 10 post-hatch. The reason is that abdominal fat, which is the main storage form of triacylglycerol in mature birds, has been reported to become clearly visible on day 7 post-hatch [24].

## 2. Materials and Methods

### 2.1. Chickens and Embryonic Heat Conditioning

All animal protocols used in this study were approved by the Institutional Animal Care and Use Committee (IACUC) at Virginia Tech. Fertile eggs from the Cobb-Hubbard chicken cross (*Gallus gallus*) were obtained from a local commercial hatchery. Upon arrival at the incubation facility, the eggs were kept at 26.6 °C for 12 h before being allocated into two groups: control and EHC, using Rite Farm Products Pro-1056 incubators. The control group was incubated at 37.8 °C with 80% humidity from day 0 to day 18.5 (E0 to E18.5), whereas the EHC group experienced a daily 12-h temperature increase to 39.5 °C with 80% humidity from E7 to E16 (07:30–19:30). At E18.5, eggs were candled to remove infertile eggs and dead embryos, and the remaining eggs were transferred to a hatcher (Rite Farm Products Pro-264) at 36.9 °C with 50% humidity for 18 h. The temperature was then gradually reduced to 35 °C until hatching. This protocol was adapted from previous work in the Cline/Gilbert laboratory [15]. Hatched chicks were weighed and randomly assigned to one of four dietary treatments: CC (control control, *n* = 17), CT (control with baicalein 500 mg/kg, *n* = 17), EC (embryonic heat control, *n* = 8), and ET (embryonic heat with baicalein 500 mg/kg, *n* = 8). The sample size for each group was based on the standard practices used in similar studies in the field. Baicalein was sourced from Xi’An Yile Bio-Tech Company, China, and its purity was confirmed by high-performance liquid chromatography (HPLC, purity ≥ 90%). The daily body weight of each chick was recorded to a tenth of a gram.

### 2.2. Tissue Collection

On day 10 post-hatch, subcutaneous and abdominal adipose tissue samples were obtained. Subcutaneous fat was collected from the junction of the breast and wing, while abdominal fat was harvested from the regions surrounding the gizzard, adjacent abdominal muscles, and the ventral side of the ischium. To access the fat pads, the skin was carefully removed, and samples were collected using sterile scissors and forceps. To ensure that no cross-contamination occurred, the workstation and instruments were thoroughly sanitized with ethanol and Kim wipes between each collection. The adipose tissue samples were promptly placed in RNALater (Fisher HealthCare, Houston, TX, USA), kept at 4 °C overnight, and subsequently stored at −20 °C until RNA isolation.

### 2.3. Total RNA Extraction and cDNA Synthesis

Adipose tissue samples (200 mg) were homogenized in TRI Reagent (Sigma-Aldrich, St. Louis, MO, USA) using 5 mm stainless steel beads (Qiagen, Valencia, CA, USA) and a Tissue Lyser II (Qiagen). Total RNA was extracted using the Zymo Quick-DNA/RNA Miniprep kit (Zymo Research, Irvine, CA, USA) according to the manufacturer’s protocol. RNA concentration and purity were assessed using a Nanophotometer Pearl Spectrophotometer (Implen, Westlake Village, CA, USA) at 260/280/230 nm. cDNA synthesis was carried out using 200 ng of total RNA and the High Capacity cDNA Reverse Transcription Kit (Applied Biosystems, Carlsbad, CA, USA) under the following reaction conditions: 25 °C for 10 min, 37 °C for 2 h, and 85 °C for 5 min.

### 2.4. Real-Time Quantitative PCR (RT-qPCR)

Primers for real-time PCR (Table 1) were designed using Primer Express (Applied Biosystems, Carlsbad, CA, USA). β-actin was used as the reference gene for mRNA expression analysis based on its stable expression in prior studies conducted in our laboratory and its widespread recognition in avian research as a reliable housekeeping gene with minimal variability across conditions. Real-time PCR was conducted in duplicate using 10 μL reaction volumes. Each reaction consisted of 5 μL of Fast SYBR Green Master Mix (Applied Biosystems, Carlsbad, CA, USA), 0.25 μL of 5 μM forward primer, 0.25 μL of 5 μM reverse primer, and 3 μL of 10-fold diluted cDNA. The reactions were performed on a 7500 Fast Real-Time PCR System (Applied Biosystems, Carlsbad, CA, USA) under the following thermal cycling conditions: an initial step of 95 °C for 20 s, followed by 40 cycles of 95 °C for 3 s, and 60 °C for 30 s. A dissociation step was included at the end of the run to verify amplicon specificity with the following profile: 95 °C for 15 s, 60 °C for 1 min, 95 °C for 15 s, and 60 °C for 15 s.

### 2.5. Plasma Non-Esterified Fatty Acid (NEFA) Collection

On day 10 post-hatch, approximately 700 µL of blood was collected from the trunk of each chick immediately following euthanasia by decapitation. Blood was collected using a funnel lined with sodium citrate anticoagulant into a 1.5 mL microcentrifuge tube lined with the same anticoagulant. Immediately after collection, the samples were vortexed and placed on ice. They were later centrifuged at 1500× *g* for 10 min using a refrigerated centrifuge at 4 °C, and the plasma was aliquoted. Plasma was stored in a −80 °C freezer until the time of assay. Plasma NEFA concentrations were measured using a 96-well flat bottom transparent plate (Applied Biosystems, Carlsbad, CA, USA) with a NEFA-HR2 kit (FUJIFILM, Wako Diagnostics, Mountain View, CA, USA) according to the manufacturer’s instructions. Absorbance was measured at 550 nm using an Infinite M200 Pro multi-mode plate reader (Tecan, Männedorf, Switzerland). Sample concentrations were calculated using the following formula:

sample concentration = standard concentration × (sample absorbance)/(standard absorbance).

The units for the concentrations are reported as mEq/L.

### 2.6. Statistical Analysis

Body weight (BW) data were analyzed using the Fit Model of JMP Pro 16 (SAS Institute Inc., Cary, NC, USA). The effects of dietary treatment, age, and their interaction were included in the BW analysis. The real-time PCR data were analyzed using the ΔΔCT method, where

ΔCT = CT target gene − CT actin, and ΔΔCT = ΔCT target sample − ΔCT calibrator.

The average of the control chicks was used as the calibrator sample. The fold difference was calculated using the 2^−ΔΔCT^ method. The PCR and NEFA results were analyzed with the Fit Model platform using JMP Pro 16 (SAS Institute Inc., Cary, NC, USA). PCR analysis included the effect of treatment, depot, and their 2-way interaction. The gene expression results were presented separately for birds under EHC and normal incubation to account for distinct environmental exposures, ensuring that the effects of each condition on gene expression are clear and interpretable.

The NEFA analysis model incorporated the primary effect of treatment. Pairwise comparisons were conducted using Tukey’s test, with statistical significance defined as a *p*-value less than 0.05.

## 3. Results

### 3.1. Growth Performance

**Figure 1:**
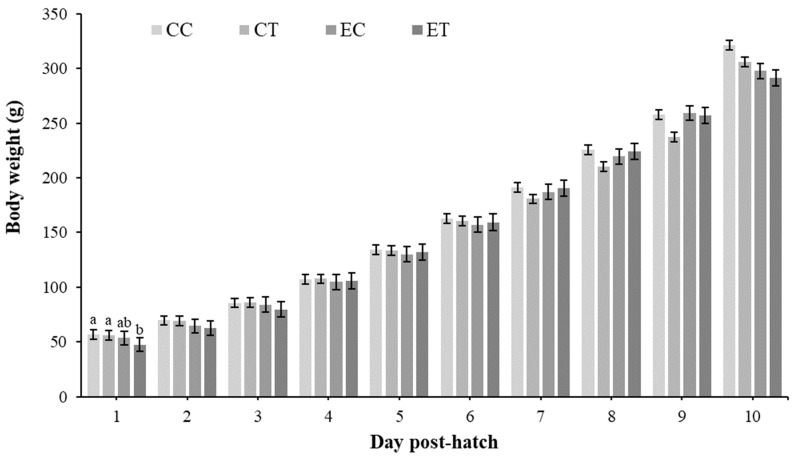
Treatment × day interaction. Day 1 *p*-value (0.0020). Values represent means ± SEM (*n* = 17 for CC and CT) (*n* = 8 for EC and ET). Different superscripts within each day indicate significant differences at *p* < 0.05; Tukey’s test. CC = control control. CT = control treatment (baicalein 500 mg/kg). EC = embryonic heat control. ET = embryonic heat treatment (baicalein 500 mg/kg).

The analysis of growth performance (Figure 1) revealed a significant difference in body weight among the treatment groups on day 1 post-hatch (*p* = 0.0020). The chicks from the ET group weighed less than those from the CC and CT groups. No significant differences were observed on the other days.

### 3.2. Adipose Tissue Relative mRNA Expression

The relative mRNA expression levels in the CC and CT groups are summarized in Table 2. The CT group showed increased mRNA expression of *C/EBPα* compared to that in the control group. However, there was no significant difference in the expression of *C/EBPβ* between the two groups. Treatment with baicalein increased the mRNA expression of *LPL*, *PPARγ*, *SREBP1*, *HSL*, *ATGL*, and *MGLL* compared to that in the control group. There were no significant differences in the mRNA expression of *DGAT2* and *NPY* between the CT and CC groups. Similarly, there was no difference in the depots, and no treatment-by-depot interaction was observed.

Table 3 shows that the ET group had decreased mRNA expression of *C/EBPα* compared to the EC group. Similarly, the mRNA expression of *C/EBPβ* was significantly lower in the ET group compared to that in the EC group. The mRNA levels of *DGAT2*, *PPARγ HSL*, and *MGLL* were significantly higher in the ET group compared to that in the EC group. The mRNA expression of *NPY* was lower in the ET group compared to that in the EC group. The expression levels of *C/EBPα*, *C/EBPβ*, and *SREBP1* were higher in the abdominal depot than in the subcutaneous depot. Depot differences were observed in the mRNA levels of *C/EBPα*, *C/EBPβ*, and *SREBP1*. No treatment-by-depot interactions were observed. PCR data are available in the Supplementary Materials section. 

### 3.3. NEFA Concentration

**Figure 2:**
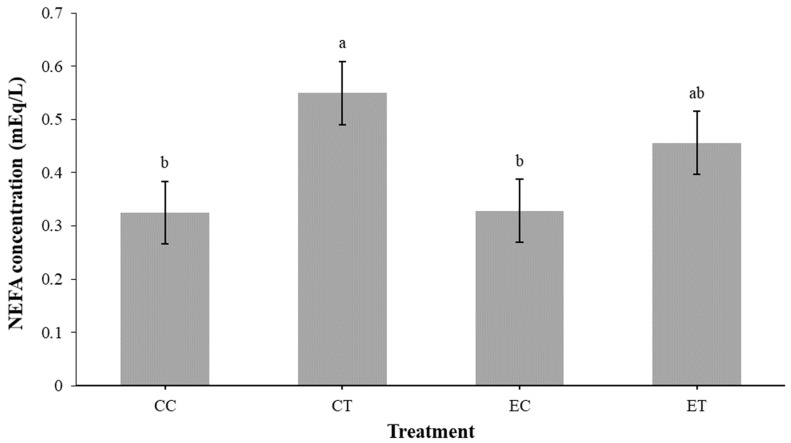
NEFA concentration by treatment on day 10 post-hatch. Values represent means ± SEM (*n*= 7). Different superscripts between treatments indicate a significant difference at *p* < 0.05, Tukey’s test. CC = control control. CT = control treatment (baicalein 500 mg/kg). EC = embryonic heat control. ET = embryonic heat treatment (baicalein 500 mg/kg).

The NEFA assay results shown in Figure 2 reveal a significant difference in NEFA concentrations among the treatment groups. Tukey’s test revealed that the mean NEFA concentrations in the CT group were significantly higher than those in the CC group. There was no difference between the EC and ET groups.

## 4. Discussion

In this experiment, we investigated the effects of dietary baicalein supplementation and embryonic heat conditioning on adipose tissue deposition in broilers. Our results indicate that these treatments impacted various aspects of adipose tissue metabolism and gene expression.

The growth performance results showed a significant difference in body weight among the treatment groups on day 1 post-hatch, with chicks from the ET group weighing less than those from the CC and CT groups. This suggests that baicalein supplementation and embryonic heat conditioning may have early effects on body weight regulation in broilers. However, no significant differences were observed on other days, indicating that the effects may be transient or specific to early post-hatch development. This is in contrast to the findings of Xiao et al. (2021), who reported a body weight reduction in Hubbard × Cobb-500 broiler chicks from days 1 to 6 post-hatch when supplemented with baicalein [22].

In this study, we chose not to statistically compare the PCR results of the control and EHC groups, as these differences have been extensively detailed in our previous publication, where we analyzed adipose tissue mRNA expression between control and EHC broilers [15]. Specifically, we reported dynamic changes in key adipogenic and lipolytic indicators, such as *C/EBPα*, DGAT, *PPARγ*, *SREBP1*, and *HSL*, in response to EHC. Here, the primary objective was to explore how baicalein differentially affects each group rather than to revisit the baseline differences between the control and EHC groups.

Analysis of the relative mRNA expression in adipose tissue revealed several findings. In the CT group, there was increased mRNA expression of *C/EBPα* and *PPARγ* compared to that in the control group. These markers are central to adipogenesis, the process by which preadipocytes differentiate into mature adipocytes. Specifically, *C/EBPα* and*PPARγ* regulate downstream processes involved in lipid metabolism and adipocyte function, orchestrating the adipogenic transcriptional cascade [25,26]. These adipogenic factors control the expression of genes required for the maturation of adipocytes [27], and the results suggest a potential role of baicalein in modulating this process. Baicalein treatment also increased the mRNA expression of key adipogenic markers, such as *LPL* and *SREBP1*, in the CT group. *LPL* is crucial for fat accumulation in mature adipocytes, as it catalyzes the hydrolysis of plasma lipoproteins, facilitating lipid transport into peripheral tissues [28]. In broiler chickens, targeting *LPL* with monoclonal antibodies has been tested as a strategy to reduce fat deposition. Results showed that antibodies effectively inhibited *LPL* activity, disrupting lipid transport into adipose tissues and consequently reducing fat accumulation [29]. *SREBP1* functions as a transcription factor that controls the expression of genes involved in de novo fatty acid and triglyceride synthesis [30]. It has been reported that the expression of *SREBP1* is positively correlated with that of *C/EBPα* and *PPARγ*, making it one of the major contributors to lipogenesis [31].

The levels of lipolytic markers also differed in this study. In fat cells, *ATGL* breaks down triglycerides (TAG) into diacylglycerol (DAG) and a free fatty acid. *HSL* then removes a fatty acid from DAG, producing monoacylglycerol (MAG). Finally, *MGLL* further breaks down MAG into a free fatty acid and glycerol [32]. In this experiment, *HSL*, *ATGL*, and *MGLL* were upregulated in the CT group compared to those in the CC group, indicating the potential of baicalein to affect fat breakdown (lipolysis). However, no significant differences were observed in the mRNA expression of *DGAT2* and *NPY* between the CT and CC groups.

In summary, baicalein supplementation was associated with increased expression of adipogenesis- and lipolysis-related markers in the CT group. A possible explanation for the increased expression of both adipogenesis and lipolytic indicators in the treatment group could be that the treatment promotes dynamic remodeling of adipose tissue. This process involves both the formation of new adipocytes and the breakdown of stored fat.

In the ET group, there was decreased mRNA expression of *C/EBPα* and *C/EBPβ* compared to that in the EC group, suggesting that embryonic heat conditioning may affect adipogenesis-related gene expression. Additionally, the mRNA levels of *DGAT2*, *PPARγ*, *HSL*, and *MGLL* were significantly higher in the ET group compared to the EC group, indicating potential effects on lipid metabolism. The mRNA expression of *NPY* was also lower in the ET group compared to the EC group, suggesting a possible role of embryonic heat conditioning in modulating appetite regulation. *NPY* is a neuropeptide known to stimulate appetite and energy storage, and its decreased expression may reflect a shift in energy balance or metabolic regulation induced by EHC [33]. Thus, similar to control chicks, gene expression results suggest that dietary baicalein supplementation during the early post-hatch period is associated with dynamic lipid remodeling in adipose tissue, with some differences in pathways as a consequence of having been exposed to the EHC.

There were also several differences in mRNA abundance across adipose tissue depots. We found that the expression levels of *C/EBPα*, *C/EBPβ*, and *SREBP1* were higher in the abdominal depot than in the subcutaneous depot of chicks incubated under control conditions. This differential expression pattern may reflect depot-specific differences in adipose tissue function and development.

Finally, the NEFA assay results showed a significant difference in NEFA levels among the treatment groups, with the CT group having higher NEFA levels than the CC group. *ATGL* breaks down the first ester bond in TAG, resulting in the release of NEFA and DAG [34]. The release of NEFAs by *ATGL* can serve as a key indicator of lipolysis, signaling the breakdown of stored fat and the mobilization of fatty acids for energy use [35]. This is consistent with the elevated *ATGL* level observed in the CT group and may suggest that baicalein supplementation may influence NEFA metabolism in adipose tissue. While adipose tissue is a major site for NEFA release during lipolysis, it is important to note that NEFAs can also originate from other tissues, such as the liver and muscles, through various metabolic processes [36]. Thus, attributing all NEFA production solely to adipose tissue would be overly simplistic. The observed differences in NEFA levels may reflect a broader systemic response to baicalein supplementation, potentially involving multiple tissues and pathways. Collectively, the NEFA results suggest that baicalein supplementation is associated with greater lipolysis, with these effects somewhat blunted in chicks that were exposed to EHC.

## 5. Conclusions

The dual action of the treatment compound—promoting both adipogenesis and lipolysis—could offer an advantage. By enhancing fat turnover, this compound could lead to leaner and more efficient broiler chickens with optimized fat levels, which is desirable for both meat quality and production efficiency. This could translate to healthier birds with improved feed conversion rates and potentially lower overall feed costs, as energy from fat is utilized more effectively. Additionally, this treatment may help reduce the risk of excessive fat deposition, which can negatively affect carcass quality and consumer preferences. Overall, our findings suggest that dietary baicalein supplementation and embryonic heat conditioning can influence adipose tissue deposition and metabolism in broiler chickens.

## Figures and Tables

**Table 1:** Primers for real-time PCR ^1^.

Gene	Sequences (Forward/Reverse)	Accession No.
β-actin	GTCCACCGCAAATGCTTCTAA/TGCGCATTTATGGGTTTTGTT	NM_205518.2
*C/EBPα*	CGCGGCAAATCCAAAAAG/GGCGCACGCGGTACTC	NM_001031459.2
*C/EBPβ*	GCCGCCCGCCTTTAAA/CCAAACAGTCCGCCTCGTAA	NM_205253.3
*DGAT2*	TTGGCTTTGCTCCATGCAT/CCCACGTGTTCGAGGAGAA	XM_040661932.1
*LPL*	GACAGCTTGGCACAGTGCAA/CACCCATGGATCACCACAAA	NM_205282.2
*PPARγ*	CACTGCAGGAACAGAACAAAGAA/TCCACAGAGCGAAACTGACATC	NM_001001460.2
*SREBP1*	CATCCATCAACGACAAGATCGT/CTCAGGATCGCCGACTTGTT	NM_204126.3
*HSL*	GCGGTGCTGAGGGAGTAC/CCCGAGACACCTCCCATAGA	XM_040657096.1
*ATGL*	GCCTCTGCGTAGGCCATGT/GCAGCCGGCGAAGGA	NM_001113291.2
*MGLL*	GCGGACGAGCGTAGACTCA/GGGAATAGCCTGGTTTGCAA	NM_001277142.2
*NPY*	CATGCAGGGCACCATGAG/CAGCGACAAGGCGAAAGTC	NM_205473.2

^1^ Abbreviations: *C/EBPα*: CCAAT/enhancer binding protein α; *C/EBPβ*: CCAAT/enhancer binding protein β; *DGAT2*: diacylglycerol O-acyltransferase 2; *LPL*: lipoprotein lipase; *PPARγ*: peroxisome proliferator-activated receptor γ; *SREBP1*: sterol regulatory element-binding transcription factor 1; *HSL*: hormone-sensitive lipase; *ATGL*: adipose triglyceride lipase; *MGLL*: monoacylglycerol lipase; *NPY*: Neuropeptide Y.

**Table 2:** Effect of baicalein on adipose tissue mRNA expression in chicks incubated under control conditions.

Effect ^1^	*C/EBPα*	*C/EBPβ*	*DGAT2*	*LPL*	*PPARγ*	*SREBP1*	*HSL*	*ATGL*	*MGLL*	*NPY*
Treatment										
CC	0.96 ± 0.12	1.03 ± 0.07	1.13 ± 0.15	0.95 ± 0.11	0.94 ± 0.07	0.96 ± 0.15	0.95 ± 0.16	1.01 ± 0.13	0.94 ± 0.11	1.16 ± 0.23
CT	1.32 ± 0.11	0.99 ± 0.07	1.14 ± 0.15	1.29 ± 0.09	1.21 ± 0.07	1.40 ± 0.14	1.49 ± 0.15	1.41 ± 0.12	1.31 ± 0.10	1.39 ± 0.23
*p*-value	0.0399	0.6095	0.4167	0.0216	0.0095	0.0374	0.0226	0.0395	0.0217	0.4773
**Depot**										
Subcutaneous	1.10 ± 0.12	0.99 ± 0.07	1.00 ± 0.15	1.08 ± 0.11	1.08 ± 0.08	1.12 ± 0.15	1.16 ± 0.17	1.15 ± 0.14	1.27 ± 0.12	1.12 ± 0.24
Abdominal	1.29 ± 0.12	1.03 ± 0.07	1.27 ± 0.15	1.20 ± 0.11	1.07 ± 0.08	1.28 ± 0.15	1.44 ± 0.17	1.32 ± 0.14	1.10 ± 0.12	1.43 ± 0.24
*p*-value	0.2783	0.6971	0.9469	0.4616	0.8957	0.4685	0.2552	0.3835	0.2901	0.3645
Treatment × Depot	0.1514	0.9899	0.2729	0.4185	0.8466	0.5333	0.1591	0.4876	0.2760	0.7104

^1^ Values represent means and standard errors of the means with associated *p*-values for the effect of baicalein dietary treatment on chicken adipose tissue depots and their interaction (*n* = 7). CC = control control. CT = control treatment (baicalein 500 mg/kg). **Abbreviation:** *C/EBPα*: CCAAT/enhancer binding protein α; *C/EBPβ*: CCAAT/enhancer binding protein β; *DGAT2*: diacylglycerol O-acyltransferase 2; *LPL*: lipoprotein lipase; *PPARγ*: peroxisome proliferator-activated receptor γ; *SREBP1*: sterol regulatory element-binding transcription factor 1; *HSL*: hormone-sensitive lipase; *ATGL*: adipose triglyceride lipase; *MGLL*: monoacylglycerol lipase; *NPY*: Neuropeptide Y.

**Table 3:** Effect of baicalein on adipose tissue mRNA in EHC chicks.

Effect ^1^	*C/EBPα*	*C/EBPβ*	*DGAT2*	*LPL*	*PPARγ*	*SREBP1*	*HSL*	*ATGL*	*MGLL*	*NPY*
**Treatment**										
EC	1.36 ± 0.11	1.13 ± 0.06	0.84 ± 0.11	0.99 ± 0.13	0.83 ± 0.08	1.31 ± 0.16	1.21 ± 0.24	1.01 ± 0.16	1.01 ± 0.13	1.28 ± 0.19
ET	0.99 ± 0.14	0.95 ± 0.06	1.23 ± 0.12	1.07 ± 0.13	1.14 ± 0.08	1.38 ± 0.16	1.92 ± 0.23	1.19 ± 0.15	1.39 ± 0.12	0.71 ± 0.20
*p*-value	0.0467	0.0150	0.0284	0.6963	0.0128	0.7538	0.0442	0.4338	0.0457	0.0500
**Depot**										
Subcutaneous	1.13 ± 0.10	0.95 ± 0.08	0.89 ± 0.12	0.99 ± 0.12	0.94 ± 0.09	1.08 ± 0.14	1.66 ± 0.36	1.08 ± 0.15	1.24 ± 0.14	1.42 ± 0.24
Abdominal	1.49 ± 0.10	1.12 ± 0.08	1.13 ± 0.13	1.07 ± 0.12	1.06 ± 0.10	1.62 ± 0.14	1.96 ± 0.36	1.14 ± 0.16	1.20 ± 0.14	0.90 ± 0.24
*p*-value	0.0167	0.0191	0.1956	0.7017	0.3760	0.0107	0.05659	0.7718	0.8551	0.1379
Treatment × Depot	0.8955	0.1710	0.6911	0.6261	0.7907	0.6097	0.3936	0.3739	0.9966	0.4167

^1^ Values represent means and standard errors of the means with associated *p*-values for the effect of embryonic heat conditioning and dietary treatment on chicken adipose tissue depots and their interactions (*n* = 7). EC = embryonic heat control. ET = embryonic heat treatment (baicalein 500 mg/kg). **Abbreviation:** *C/EBPα*: CCAAT/enhancer binding protein α; *C/EBPβ*: CCAAT/enhancer binding protein β; *DGAT2*: diacylglycerol O-acyltransferase 2; *LPL*: lipoprotein lipase; *PPARγ*: peroxisome proliferator-activated receptor γ; *SREBP1*: sterol regulatory element-binding transcription factor 1; *HSL*: hormone-sensitive lipase; *ATGL*: adipose triglyceride lipase; *MGLL*: monoacylglycerol lipase; *NPY*: Neuropeptide Y.

## Data Availability

The original contributions presented in this study are included in the article/Supplementary Materials. Further inquiries can be directed to the corresponding author(s).

## Supplementary Materials

The following supporting information can be downloaded at https://www.mdpi.com/article/10.3390/ani14243563/s1, PCR Data.
